# The Clinical Outcome of Laparoscopic Surgery for Endometriosis on Pain, Ovarian Reserve, and Cancer Antigen 125 (CA-125): A Cohort Study

**DOI:** 10.22074/IJFS.2021.137035.1018

**Published:** 2021-10-16

**Authors:** Fereshte Sarbazi, Elham Akbari, Anita Karimi, Behnaz Nouri, Shahla Noori Ardebili

**Affiliations:** 1 Department of Obstetrics and Gynaecology, Farmanieh Hospital, Tehran, Iran; 2 Department of Obstetrics and Gynaecology, Shahid Beheshti University of Medical Sciences, Shohadaye Tajrish Hospital, Tehran, Iran

**Keywords:** Anti-Mullerian Hormone, CA-125 Antigen, Endometriosis, Pain, Patient Outcome Assessment

## Abstract

**Background:**

Endometriosis is an important cause of chronic pain and infertility. Surgery is considered the gold standard for diagnosis and treatment. In this study, we aim to describe the clinical outcomes of women who undergo laparoscopic surgery for endometriosis.

**Materials and Methods:**

In this cohort study, a total of 174 women who referred to Farmaniyeh Hospital, Tehran, Iran from August 2015 to December 2017 with surgical diagnoses of endometriosis stages III and IV enrolled. The participants’ demographic, gynaecological, and clinical characteristics were recorded and they were asked to use a numeric rating scale (NRS) to record their severity of pain before and three months after surgery. Blood samples were also taken from the patients before and three months after surgery for measurement of serum levels of anti-Müllerian hormone (AMH) and cancer antigen 125 (CA-125). Data were analysed using SPSS version 21.

**Results:**

The patients had a mean age of 34.86 ± 6.47 years, 60.9% were married, and 49.4% were housewives. The primary indication for surgery was pain (68.4%), followed by both pain and infertility in the remainder of patients. Types of endometriotic lesions included endometrioma (19%), deep infiltrating endometriosis (DIE, 3.4%), and both endometrioma and DIE (77.6%). There was a reduction in pain from 6.79 ± 2.19 before surgery to 1.48 ± 1.68 after surgery; serum AMH levels reduced from 2.80 ± 1.86 ng/mL to 1.76 ± 1.40 ng/mL and CA- 125 reduced from 257.06 ± 220.25 U/mL to 23.27 ± 23.25 U/mL (all P<0.001). Of the 21.2% who experienced recurrence, 13.5% underwent additional surgery. The total additional surgery rate was 2.8%. Of the 55 patients with infertility, 78.1% became pregnant after surgery, 54.5% of which was spontaneous.

**Conclusion:**

Surgical treatment of endometriosis had a favourable effect on the patients’ pain and inflammation and resolved the patients’ infertility with a minimal need for additional surgery.

## Introduction

Endometriosis is a benign gynaecologic disorder mainly observed in reproductive age women that has a global prevalence of 5-15% (1). The most common symptoms of endometriosis include chronic pelvic pain, dysmenorrhea, menorrhagia, dyspareunia, gastrointestinal (GI) complaints, and urinary symptoms (2); some cases may remain asymptomatic or may have mild symptoms, whereas others may only present with disease complications such as chronic pelvic pain and infertility (3). The disease severity of endometriosis varies based on the lesion site and penetration of the endometriotic lesions into the peritoneum; deep infiltrating endometriosis (DIE) has the worst prognosis and most severe symptoms (4).

Several hypotheses have been proposed for the pathogenesis of endometriosis; however, the majority of patients’ symptoms appear to be associated with inflammation and proliferation of endometriotic lesions (5) that result from the secretion of cytokines and growth factors such as tumour necrosis factor-alpha (TNF-α) and interferon-gamma (IFN-γ), and cancer antigen 125 (CA-125, used for diagnosis of endometriosis) (6). Anti-Müllerian hormone (AMH) is a member of the TGF-β superfamily and considered a valuable serum marker for a general measurement of ovarian reserve and also in women with endometriosis (7). 

The clinical symptoms of endometriosis are not specific and serum/peripheral biomarkers can only predict endometriosis (8); therefore, direct observation of lesions during surgery and histologic confirmation of the specimens are considered the gold standard for diagnosis and treatment (9). A six-year follow-up of 1315 patients confirmed that laparoscopic treatment of endometriosis resulted in significant reductions in pain and resolution of infertility, which resulted in pregnancy after surgery (spontaneous or assisted) (10). Due to the need for ongoing reporting and more detailed follow-up after surgery for endometriosisassociated pain, in this study we aimed to describe the clinical characteristics of women who underwent laparoscopic surgery for treatment of endometriosis as well as the effect of surgery on patients’ pain and serum levels of AMH and CA-125.

## Materials and Methods

### Study design

In this cohort study, all women who referred to Farmaniyeh Hospital in Tehran, from August 2015 to December 2017 for surgical treatment of endometriosis enrolled in this study. Diagnosis of endometriosis was based on the clinical symptoms of endometriosis and the results of imaging tests (ultrasound and magnetic resonance imaging [MRI]) in patients with severe pain or both pain and infertility who were indicated for surgical treatment. Those with surgically confirmed endometriosis stages III or IV were included in this study by the census method, after they received explanations about the study objectives and read and sign the written informed consent form. The Ethics Committee of Farmaniyeh Hospital approved the study protocol (FH-02- 005). This study was conducted in accordance with the principles of the Declaration of Helsinki and its subsequent amendments.

The surgeries for all enrolled patients were performed by one surgical team of two gynaecologic laparoscopic surgeon.

For this purpose, after induction of general anaesthesia by the anaesthesiologist, the trocars were inserted in their place and the abdomen and pelvic cavity were explored. After visualization of the endometrioma, the cyst wall was excised and the ovarian adhesions were released to mobilize the ovaries, and the DIE lesions were totally resected. 

The researcher used a study checklist to record the participants’ demographic characteristics (age, height, weight, body mass index [BMI], marital status, educational level, and occupational status), gynaecological characteristics (age at menarche and menopause, menorrhagia, metrorrhagia, dysmenorrhea, dyspareunia, regular or irregular menstruation, history of endometriosis surgery, history of infertility before surgery, and indication for surgery), and endometriotic-related characteristics (type of endometriotic lesion, presence of endometrioma, its type and side, and the anatomical site of the DIE). The information was collected from the hospital medical records and completed by conferring with the patients (history taking). Cases of recurrence, reoperation, and pregnancy after surgery with or without assisted reproductive technique (ART) were recorded. Follow-up information was collected during post-surgical follow-up visits or by phone contact with patients who did not return for their follow-up visits. Recurrence was defined as recurrence of endometrioma or pain. Cases with recurrence were treated by medical therapy and surgery, if required.

The participants were asked to record their pain severity before and three months after surgery on a numeric rating scale (NRS), which was scored from 0 to 10 where 0 indicated no pain and 10 indicated the worst pain. Two blood samples were taken from the patients, one before surgery and three months after surgery. The samples were sent to the laboratory for measurement of serum levels of AMH and CA-125 antigen. The tumour markers were measured using an Enzyme linked Fluorescent Assay (ELFA) technique (TOSOH Co.) and CA-125 levels <35 were considered normal. AMH was measured using ELISA kits (Beckman Coulter Co., USA). AMH levels of 4.0-6.8 indicated optimal fertility, 2.2-4.0 indicated satisfactory fertility, 0.3-2.2 indicated low fertility, and <0.3 indicated very low fertility.

Cases who were not confirmed as having endometriosis by surgical inspection were excluded from the study and not included in the statistical analysis. 

### Statistical analysis

The results were described by frequency (%) for categorical variables and by mean ± standard deviation (SD) for quantitative variables. The results of the Kolmogorov-Smirnov test showed normal distribution of the data; therefore, we used the paired t test to compare numeric variables before and after surgery. The chi square test was used to compare frequencies between the groups. For statistical analysis, we used the IBM SPSS Statistics for Windows version 21.0 (IBM Corp., Armonk, NY, USA.) statistical software. P<0.05 were considered statistically significant. 

## Results

The 174 women who completed the study had a mean age of 34.86 ± 6.47 (18-49) years. Most (60.9%) were married, about half (49.4%) were unemployed/ housewives, and 64.3% had an academic education. Table 1 lists the demographic and gynaecologic characteristics of the participants. The indication for surgery was pain in the majority (68.4%) of patients and both pain and infertility in the rest.

Types of endometriotic lesions included: endometrioma (19%, n=33), DIE (3.4%, n=6), and both endometrioma and DIE (77.6%, n=135); 77.4% of cases with endometrioma were bilateral (n=130) and 22.6% (n=38%) were unilateral. The frequency of endometrioma located on the left side (57.9%) was significantly higher than the right side (42.1%, P=0.003), but the frequency of DIE on the left or right sides did not have any significant difference (47.6 vs. 52.4%, respectively, P=0.371). As shown, the most common site of DIE was the ovarian fossa and the least common site was the vaginal vault (1.19%).

**Table 1:** Demographic and gynaecologic characteristics of the study population


Variable		n	Mean ± SD or %

Age (Y)		174	34.86 ± 6.47
Weight (kg)		173	68.12 ± 13.88
Height (m)		173	164.90 ± 5.53
BMI (kg/m^2^)		173	24.95 ± 4.40
Menarche age (Y)		133	12.15 ± 1.48
Menopause age (Y)		7	45.71 ± 1.25
Menorrhagia		44	25.3
Metrorrhagia		36	20.7
Dysmenorrhea		164	95.9
Dyspareunia		101	58.0
Irregular menstruation		42	24.1
Marital status			
	Single	68	39.1
	Married	106	60.9
Educational level			
	Illiterate	16	9.2
	High school graduate	34	19.5
	B.Sc.	75	43.1
	M.Sc.	22	12.6
	Ph.D.	15	8.6
	Not reported	12	6.9
Occupational status			
	Housekeeper/unemployed	86	49.4
	Employed	85	48.9

Previous surgery for endometriosis		24	13.8
Indication for surgery			
	Pain and infertility	55	31.6
	Pain	119	68.4


SD; Standard deviation and BMI; Body mass index.

**Fig.1:**
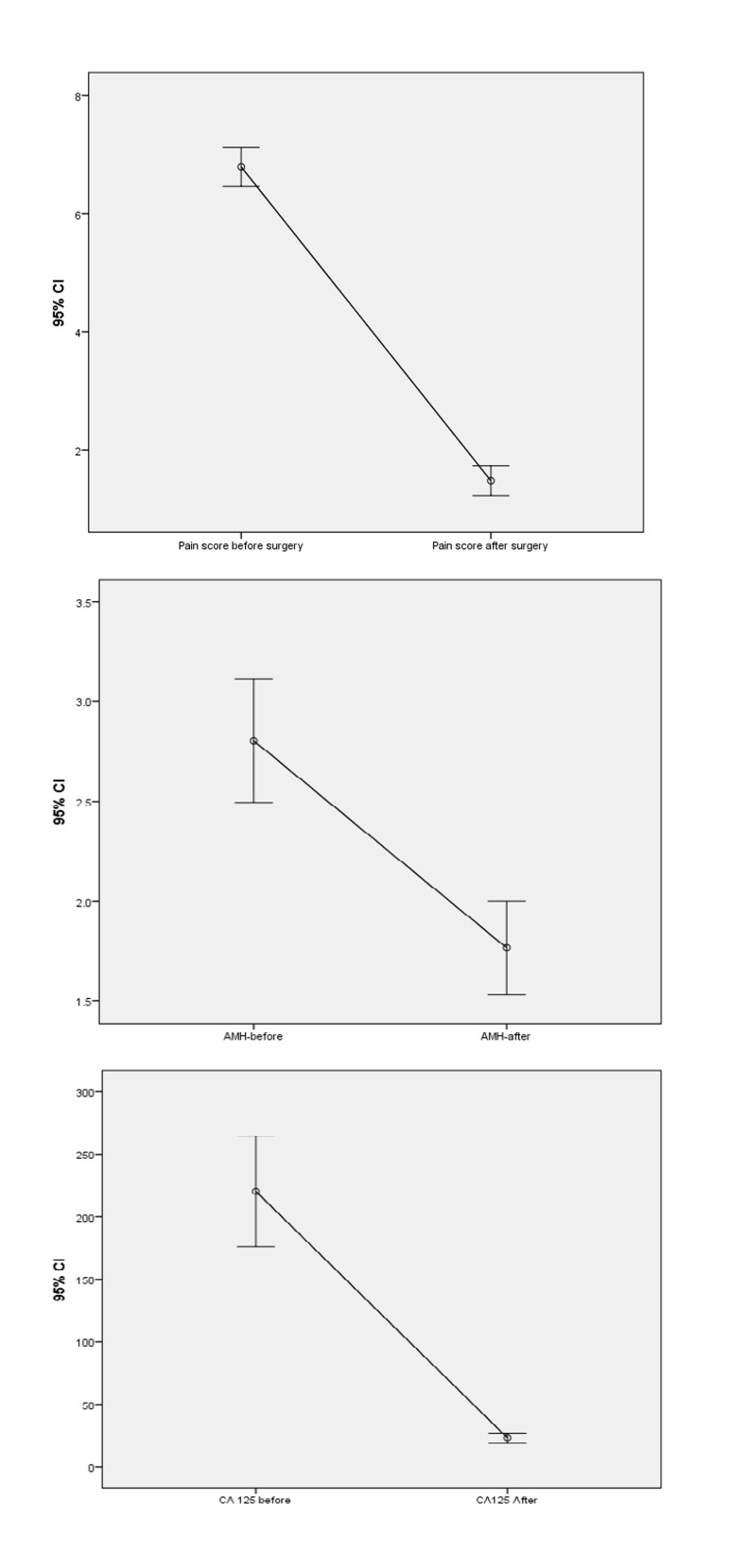
The changes in mean scores of pain, and serum levels of anti-Müllerian hormone (AMH) and cancer antigen 125 (CA-125) post-surgery compared to pre-surgery. CI; Confidence interval.

Participants reported a reduction in pain from 6.79 ± 2.19 before surgery to 1.48 ± 1.68 after surgery; in addition, serum levels of AMH reduced from 2.80 ± 1.86 ng/mL before surgery to 1.76 ± 1.40 ng/mL after surgery and that of CA-125 from 220.25 ± 257.06 U/mL before surgery to 23.27 ± 23.25 U/mL after surgery (all P<0.001, Table 2). Figure 1 shows the trend of changes in pain, AMH, and CA-125. Postoperative follow-up showed recurrence in 21.2% of patients and 13.5% of these patients underwent additional surgery. The total reoperation rate was 2.8%. Of 55 patients who had a positive history of infertility, 43 (78.1%) became pregnant after surgery, 54.5% of these were spontaneous and without ART, and 23.6% with the use of ART.

**Table 2:** The post-surgical outcome of the study participants


Post-surgical outcome		n	Mean ± SDor (%)	P value

Pain score before surgery		173	6.79 ± 2.19	<0.001
Pain score after surgery		173	1.48 ± 1.68	
AMH before surgery (ng/mL)		142	2.80 ± 1.86	<0.001
AMH after surgery (ng/mL)		142	1.76 ± 1.40	
CA-125 before surgery (U/mL)		133	220.25 ± 257.06	<0.001
CA-125 after surgery (U/mL)		133	23.27 ± 23.25	
Recurrence		37	21.2	–
Reoperation (% of relapsed cases)		5	13.5	–
Reoperation (% of total cases)		5	2.8	
History of infertility before surgery		55	31.6	–
Pregnancy after surgery				
	Without ART (% of infertile cases)	30	54.5	-
	With ART (% of infertile cases)	13	23.6	–


SD; Standard deviation, AMH; Anti-Müllerian hormone, CA-125; Cancer antigen 125, and ART; Assisted reproductive technique.

## Discussion

In this study, we described the characteristics of 174 women with endometriosis, indicated for surgical treatment, and reported the follow-up results of these patients. The age range of 18 to 49 years (mean age: 34.86 years) and menopause in only seven of our patients confirmed the main occurrence of endometriosis in women of reproductive age and its rare occurrence after menopause (1). The mean age of menarche in the present study (12.15 years) also confirmed the results of a meta-analysis of 47 Iranian studies (11). 

Endometrioma and DIE were present in 96.6% and 81% of patients, respectively, alone or in combination. The high frequency of coexistence of endometrioma and DIE refers to their association, as reported previously (12). Furthermore, as our patients were surgical candidates, the high frequency of endometrioma and DIE confirmed the association of these two with increased disease severity, as suggested previously (13). Most cases of endometrioma were bilateral (77.4%) and the frequency of left-sided endometrioma was higher. A higher frequency of endometriosis on the left side is anticipated to result from the anatomical differences of abdomen (presence of diaphragm on the right side) and hemipelvis (presence of sigmoid colon on the left side) (14). Right and left ovarian fossa were also the most common sites of DIE in our study, which was in line with evidence that suggested an association of ovarian involvement with more severe DIE (15).

The primary endometriosis symptoms in our patients included dysmenorrhea (95.9%) and dyspareunia (58%), while menstruation-related problems such as menorrhagia, metrorrhagia, and irregular menstruation had a frequency of 20-25%. Different frequencies have been reported in women with endometriosis (16, 17). However, of note is the significance of different forms of pain (dysmenorrhea and dyspareunia) in women with endometriosis in our study that referred to the importance of endometriosisassociated pain, which was in line with the results of previous studies that referred to the important role of pain and the impact of endometriosis on a woman’s life (18). Pain was the indication of surgery in all patients (with or without infertility) and assessing the pain severity in our patients showed a high severity of pain in women before surgery (mean: 6.79), which confirmed the significance of pain in these patients. After surgery, a significant decrease was observed in patients’ pain. In the study by Alborzi et al. (10), a six-year follow-up of patients showed reduced pain scores from 8.23 to 4.46 in 93.07% of patients. The differences in the scores could be due to the different evaluations of the patients from pain, as the assessment tool is a self-report tool and due to the different surgical details used because of the different sites and penetrations of endometriotic lesions. A meta-analysis of 1847 patients (23 studies) also showed significant reductions in pain after endometriosis surgery with a decrease of approximately 4.5-5.2 units in the pain scores, which differed based on the duration of the follow-up period (19). These results also confirmed the present study findings and suggested that the endometriotic lesions caused inflammation and activated the central nervous system (CNS). Therefore, excision of these lesions resolved these problems and the resulting pain (20). However, it has been reported that some cases may recur over time (21). In our study, we observed recurrence in 21.2% cases and a total of 2.8% required additional surgery. These results were in line with that reported by Asadzadeh and colleagues on an Iranian population where 28.6% of cases recurred after surgery (22). The different surgical details and the difference in the frequency of lesions’ sites might affect the recurrence rate. 

Another important aspect of endometriosis is infertility. In the present study, the majority of our patients were married and infertility was observed in more than half of the married cases (51.8%) and about one-third of all cases (31.6%), which referred to the significance of infertility in women with endometriosis. The follow-up in our study showed that 78.1% of infertile women became pregnant after surgery, 54.5% were spontaneous and 23.6% after ART. In a study by Alborzi et al. (10), 58.1% of infertile women became pregnant during the follow-up period, which was lower than the present study results. Furthermore, the frequency of infertility was also lower in their study (about 15%) compared to the current study. Infertility is one of the indications of surgical treatment for endometriosis and one of the main goals of the surgical treatment (23). Therefore, it is necessary to pay more attention to this issue. Depletion of ovarian reserve is reported to be one of the causes of endometriosis-related infertility (24). Hence, AMH, levels are commonly measured for assessment of ovarian reserve and it is also suggested to be measured in women with endometriosis (25). In the present study, although a statistically significant reduction was observed in serum levels of AMH, both pre- and post-surgical values were within the normal range. These results were in line with a report by Streuli and colleagues, which suggested that endometriosis did not result in decreased serum AMH levels and low AMH levels were only observed in patients with surgical histories of endometriosis (26). Others have also reported a decline in serum AMH levels after surgery (27) that was associated with bilaterality and disease severity (28), which confirmed the results of the present study.

As a reliable marker for diagnosis of endometriosis, CA-125 was also measured in the present study and the results showed that the significantly high mean pre-surgical level of CA-125 reduced after surgery. These results confirmed the previous study results, considering the high level of CA-125 in peritoneal fluid and sera of patients with endometriosis (8) and significant reduction (25.8%) in CA-125 levels after surgery (29). This high molecular weight glycoprotein, produced in the epithelium, can be a good marker for diagnosis and follow-up of endometriosis. One of the limitations of the present study was the small sample size and nonrandomized patient enrolment. Furthermore, the pain assessment tool is a self-report tool and is exposed to subjective bias. Because of the multifactorial nature of endometriosis, the effect of confounders on the study results cannot be rejected; however, we evaluated a wide range of variables to overcome this issue.

## Conclusion

The results of the present study showed that surgical treatment of endometriosis could have a favourable effect on patients’ pain and infertility with a minimal rate of reoperation and acceptable recurrence rate. However, the role of AMH in endometriosis and the serum level of CA-125 after surgery should be studied in future research.
